# Aqueous humor level of sCD44 in patients with degenerative myopia and primary open-angle glaucoma

**DOI:** 10.1186/1756-0500-2-224

**Published:** 2009-11-08

**Authors:** Yasemin U Budak, Muberra Akdogan, Kagan Huysal

**Affiliations:** 1 Department of Clinical Laboratory, Sevket Yilmaz Hospital, Bursa, Turkey; 2 Department of Ophthalmology, Sevket Yilmaz Hospital, Bursa, Turkey; 3 Department of Clinical Laboratory, Yuksek Ihtisas Training Hospital, Bursa, Turkey

## Abstract

**Background:**

The transmembrane glycoprotein CD44 is a major hyaluronan cell surface receptor widely distributed in eye tissues and fluids. The shed ectodomain of CD44 is termed soluble CD44 and is toxic to human retinal ganglion cells in cell culture. The purpose of this study was to investigate the concentration of sCD44 in the aqueous humor (AH) of normal subjects, patients with primary open-angle glaucoma, and patients with degenerative myopia but without glaucoma, to determine if the molecule might serve as a protein marker of glaucoma.

**Findings:**

In this case-control study, AH samples were collected from controls (n = 16), patients with primary open-angle glaucoma (n = 11), and patients with degenerative myopia (n = 11) who underwent phacoemulsification surgery to treat mature or immature cataracts. The sCD44 concentration in AH was measured using a commercial ELISA kit.

In normal AH samples the sCD44 concentration was 5.40 ± 1.21 ng/mL, whereas in degenerative myopia patients the sCD44 concentration was 5.76 ± 1.15 ng/mL. There was thus no statistically significant difference between these two groups (p > 0.05). The aqueous sCD44 concentration in patients with primary open-angle glaucoma (12.2 ± 10.1 ng/mL) was higher than that of the control group (p < 0.05).

**Conclusion:**

sCD44 may be a protein marker of primary open-angle glaucoma.

## Background

Glaucoma, a leading cause of visual impairment and blindness worldwide, is characterized by excavation of the optic nerve head and selective apoptotic loss of retinal ganglion cells (RGCs), resulting in a progressive decline in visual function [1]. There are several types of glaucoma (includingopen-angle, normal tension, and early-onset). Primary open-angle glaucoma (POAG) is the most common form of disease. The major risk factor for most glaucomas and POAG is increased intraocular pressure [1]. Intraocular pressure is a function of the production of liquid aqueous humor (AH) by the ciliary processes of the eye and drainage thereof through the trabecular meshwork (TM). Myopia, or short-sightedness, is the most common form of vision disorder worldwide. Higher levels of myopia, usually defined as an axial eye length >26 mm or a refractive error of < -6.00 diopters (D), are often designated as 'degenerative' myopia. Patients with high levels of myopia (spherical equivalent at least -6.0 D) are more susceptible to glaucoma [2].

The majority of aqueous outflow resistance in both normal and glaucomatous eyes lies within the TM, especially within the extracellular matrix of the juxtacanalicular connective tissue, which is a glycosaminoglycan-enriched area [3]. The transmembrane glycoprotein CD44 is a major hyaluronic acid (HA) cell-surface receptor widely distributed in eye tissues and fluids [4]. CD44 functions as a platform for growth factors and membrane-associated matrix metalloproteinases (MMPs), thereby entrapping molecules affecting growth, and bringing enzymes and substrates together [5]. In addition, CD44 is required for activation of certain high-affinity receptors (*e.g*., those involved in erbB2 phosphorylation and erbB2-erbB3 heterodimerization) required for cell survival [6]. Proteolytic cleavage of the extracellular domain of CD44 by MMPs releases sCD44, which has biological functions that differ from those of the intact CD44 protein [7]. It has recently been shown that sCD44 adversely affects retinal ganglion cells and TM cell survival *in vitro*, by activating a proapoptotic pathway [8].

The purpose of the present study was to investigate the concentration of sCD44 in the AH of normal subjects, and patients with POAG and degenerative myopia without glaucoma, to determine if this molecule might serve as a protein marker of glaucoma.

## Patients and Methods

The patients enrolled in this study were derived exclusively from the Turkish population. Eleven POAG patients (six male, five female), 11 degenerative myopia patients (four male, seven female) who underwent phacoemulsification surgery to treat mature or immature cataracts, and 16 normal patients (eight male, eight female), were selected for this case-control study. All subjects underwent a systematic examination of the optic disc, visual field examination with automated static white-on-white threshold perimetry using the 30-2/24-2 program of the Octopus Visual Field Analyzer (Interzeag, Schlieren, Switzerland), and IOP measurement employing Goldmann applanation tonometry (Haag Streit, Bern, Switzerland).

AH specimens from anterior chambers were obtained during phacoemulsification surgery. Exclusion criteria included prior incisional ocular surgery, other ophthalmic diseases (for example, uveitis or progressive retinal disease), and diabetes mellitus. Patients provided informed consent after the nature and consequences of the study were explained, in accordance with the tenets of the Declaration of Helsinki.

An attempt was made gather AH samples under uniform conditions. Thus, the duration of body rest in the supine position in the operating theatre, and the temperature of the theatre, were standardized. A small amount of AH (50 μL) was aspirated using a 27-gauge needle on a tuberculin syringe, with special care to avoid blood contamination. Immediately after collection, aqueous samples were stored at -20°C until analysis. Samples that could not be evaluated within 4 months of storage were not included in this study. The aqueous concentration of sCD44 was measured using a commercial ELISA kit (Bender MedSystems, Wien, Austria).

### Statistical analysis

Means and standard deviations were calculated and we performed nonparametric Mann-Whitney U tests to compare sCD44 concentrations in normal AH, POAG AH, and degenerative myopia AH samples. A p value < 0.05 was considered significant.

## Results

The clinical data from patients with POAG, and degenerative myopia without glaucoma, compared with those of normal subjects, are summarized in Table 1. In normal aqueous samples the sCD44 concentration was 5.40 ± 1.21 ng/mL, and in degenerative myopia patients the concentration was 5.76 ± 1.15 ng/mL. There was no statistically significant difference between these two groups (p > 0.05). The aqueous sCD44 concentration of patients with POAG (12.2 ± 10.1 ng/mL) was higher than that of the control group (p < 0.05).

**Table 1:** The clinical parameters of age, gender, refractive error, duration of POAG, cup-disc ratio in each of the cohorts (control, POAG including duration of POAG, and myopia)

	Control	POAG	Degenerative myopia
**Age**	65 ± 8	63 ± 10	57 ± 11
**Gender**	8 M/8 F	6 M/5 F	4 M/7 F
**Refractive error**	+0.9 ± 0.18	+1.1 ± 1.5	-14.3 Dsph ± 7.0
**Average Cup/disc ratio**	0.47 ± 0.20	0.60 ± 0.26	0.40 ± 0.20
**Duration of POAG**		11 ± 6	

## Discussion

AH is an important intraocular fluid responsible for the supply of nutrients to and removal of metabolic wastes from the avascular tissues of the eye [9]. AH contains proteins secreted from anterior segment tissues, and these proteins could play roles in the pathogenesis of various eye diseases [10]. Several studies have demonstrated that changing levels of certain AH proteins correlate with the mechanism or prognosis of many eye disorders [11-13]. In the present study, we directed our attention to AH collected directly from patients. Despite the small number of participants, we found that the sCD44 level was increased, with statistical significance, in the AH of POAG patients compared with normal subjects and degenerative myopia patients without glaucoma. The sCD44 level was only slightly elevated in degenerative myopia patients, but this difference was not statistically significant. It is nonetheless possible that a study with a larger patient series would show elevated sCD44 in the AH of such patients. A similar observation was reported by Knepper and colleagues, who found a significant increase in AH sCD44 level in POAG and normal-tension glaucoma patients [14,15].

Only a few studies have explored the functions and clinical importance of aqueous sCD44;both aspects thus remain poorly understood. According to previous reports, the bioavailability of sCD44 depends on binding to HA, and the extent of such binding is influenced by pressure. HA has hydrophobic patches affected by elevated pressure, as demonstrated by electron microscopy and rotary shadowing [16]. In normal AH, HA binds to and inactivates sCD44 [17]. In POAG TM and AH, HA concentration is decreased and AH sCD44 level is twice the normal value [7,14,18]. Once the concentration of sCD44 attains a particular threshold, the molecule becomes cytotoxic to some target cells (*e.g*., TM cells, RGCs, or supporting cells in the prelaminar portion of the optic nerve) [17]. Knepper and co-workers hypothesized that the apparent change in the HA polymer with increased pressure, and the decreased binding of sCD44 to HA under such circumstances, may partly explain why increased IOP is a clinical risk factor for POAG [17].

In the present study, sCD44 concentration in POAG AH showed wide variation. It is known that POAG is a genetically heterogeneous disorder, featuring interaction of multiple genes and affected by environmental influences [19]. We suggest that the increase in sCD44 concentration in POAG AH is secondary to an underlying heterogeneous pathophysiology and that AH levels of sCD44 may be influenced by other factors.

In a previous study, Nolan and associates examined correlations between AH sCD44 concentration and known POAG risk factors [20]. In the cited work, sCD44 concentrations in POAG patients with myocilin mutations were lower than in other POAG patients. The cited authors also found that filtration surgery significantly reduced the CD44 level in POAG patients [20]. Because our POAG group was small and heterogeneous, the data are insufficient to permit subgroup analysis of our patient group.

Another explanation for the wide variation in sCD44 concentration seen in POAG AH might be that we were unable to obtain POAG AH from patients who had not been treated with glaucoma drugs. Nolan et al. showed that use of carbonic anhydrase inhibitors tended to increase sCD44 levels in AH. On the other hand, the use of prostaglandin inhibitors, which enhance AH outflow, tended to decrease sCD44 concentration [20].

Myopic subjects are at a two- to three-fold increased risk of glaucoma compared with nonmyopic subjects. The risk was independent of other glaucoma risk factors and IOP [21]. Recently, Li and colleagues found that the concentration of HA was lower in the vitreous of high-myopia patients [22]. Highly myopic eyes show markedly reduced amounts of the markers collagen and glycosaminoglycans, when compared with the sclera of emmetropic eyes [23]. To the best of our knowledge, this is the first study measuring sCD44 levels in degenerative myopic AH.

A significant limitation of the present study was the lack of a patient group with both high-level myopia and glaucoma. Further investigation is necessary to understand the exact relationship between AH changes leading to glaucoma in high-myopia patients.

The results of the present study reveal that the sCD44 level in AH differed significantly between the POAG and control group. Although our sample size was small, statistical significance was attained, and we thus suggest that sCD44 may serve as a protein marker of POAG. However, a study with a larger number of patients is needed to confirm the validity of this observation. sCD44 was slightly, but not significantly, elevated in the AH of degenerative myopia patients. Analysis of a larger patient series might reveal that such patients do in fact have elevated sCD44, but perhaps to an extent lower then seen in the AH of POAG patients.

## Competing interests

The authors declare that they have no competing interests.

## Authors' contributions

All authors have made substantial contributions to design of the work, in addition to analysis and interpretation of data; and have been involved in drafting the article and revising it critically for important intellectual content; and have given final approval of the version to be published.
